# Literary Note

**Published:** 1908-09

**Authors:** 


### LITERARY NOTE

THE Philadelphia Academy of Surgery announces that for the Samuel D. Gross prize of $1,500. essays will be received in competition until January 1, 1910. The conditions annexed by the testator are that the prize "Shall be awarded every five years to the writer of the best original essay, not exceeding one hundred and fifty pages, octavo, in length, illustrative of some subject in surgical pathology or surgical practice, founded upon original investigations, the candidates for the prize to be American citizens."

It is expressly stipulated that the competitor who receives the prize, shall publish his essay in book form, and that he shall deposit one copy of the work in the Samuel D. Gross Library of the Philadelphia Academy of Surgery, and that on the title page, it shall be stated that to the essay was awarded the Samuel D. Gross Prize of the Philadelphia Academy of Surgery.

The essays, which must be written by a single author in the English language, should be sent to the "Trustees of the Samuel D. Gross Prize of the Philadelphia Academy of Surgery, care of the College of Physicians, 219 S. 13th St., Philadelphia," on or before January 1, 1910.

